# Transpancreatic Sphincterotomy Is a Safe and Effective Pancreatic Guidewire-Assisted Cannulation Method: Real-World Data Analysis of the Hungarian ERCP Registry

**DOI:** 10.3390/jcm14197118

**Published:** 2025-10-09

**Authors:** Dániel Pécsi, Nelli Farkas, Szilárd Gódi, Péter Hegyi, Andrea Szentesi, István Altorjay, Tamás Bakucz, Ákos Orbán-Szilágyi, Zoltán Szepes, László Czakó, Árpád Patai, Tibor Gyökeres, Roland Fejes, Zsolt Dubravcsik, Áron Vincze

**Affiliations:** 1 Institute for Translational Medicine, Medical School, University of Pécs, 7624 Pécs, Hungary; pecsi.daniel@pte.hu (D.P.); nelli.farkas@aok.pte.hu (N.F.); hegyi.peter@pte.hu (P.H.); szentesia@gmail.com (A.S.); 2 Division of Interventional Gastroenterology, First Department of Medicine, Medical School, University of Pécs, 7624 Pécs, Hungary; godi.szilard@pte.hu; 3 Institute for Bioanalysis, Medical School, University of Pécs, 7624 Pécs, Hungary; 4 Centre of Translational Medicine, Semmelweis University, 1085 Budapest, Hungary; 5 Gastroenterology Clinic, University of Debrecen, 4032 Debrecen, Hungary; altorjay@med.unideb.hu; 6 Department of Gastroenterology, Central Hospital of Northern Pest—Military Hospital, 1134 Budapest, Hungary; bakucz.tamas@gmail.com (T.B.); szakoos@gmail.com (Á.O.-S.); tiborgyokeres65@gmail.com (T.G.); 7 First Department of Medicine, University of Szeged, 6720 Szeged, Hungary; szepes.zoltan@med.u-szeged.hu (Z.S.); czako.laszlo@med.u-szeged.hu (L.C.); 8 First Department of Gastroenterology and Medicine, Markusovszky University Teaching Hospital, 9700 Szombathely, Hungary; pataiarpaddr@gmail.com; 9 First Department of Medicine, Szent György University Teaching Hospital of County Fejér, 8000 Székesfehérvár, Hungary; rolldoc@vipmail.hu; 10 Bács-Kiskun County University Teaching Hospital, 6000 Kecskemét, Hungary; dubravcsikzs@gmail.com

**Keywords:** endoscopic retrograde cholangiopancreatography, advanced cannulation, biliary cannulation, adverse events, transpancreatic sphincterotomy, Post-ERCP pancreatitis

## Abstract

**Background/Objectives:** Difficult biliary cannulation during endoscopic retrograde cholangiopancreatography (ERCP) poses significant challenges and increases the risk of adverse events. Pancreatic guidewire (PGW)-assisted techniques offer potential solutions, but real-world comparative data are limited. **Methods:** This cohort study of prospectively collected data analyzed 234 ERCP cases from the Hungarian ERCP Registry, focusing on three PGW-assisted methods: transpancreatic sphincterotomy (TPS), double-guidewire technique (DGW), and prophylactic pancreatic stent-assisted cannulation (PPS-C). **Results:** TPS demonstrated the highest primary cannulation success rate (83.1%), significantly outperforming DGW (67.7%) and PPS-C (67.6%) (*p* < 0.001). With salvage methods, cannulation success was high across all groups. Post-ERCP pancreatitis rates were low (5.0% TPS, 5.6% DGW, 3.9% PPS-C), but prophylactic measures (pancreatic stents, indomethacin) were underutilized. **Conclusions:** Our findings suggest that TPS is a safe and effective alternative for difficult biliary cannulation in ERCP. Routine considerations of post-ERCP pancreatitis prophylaxis (prophylactic pancreatic stents and non-steroidal suppositories) are recommended in all PGW-assisted cannulations to minimize complications.

## 1. Introduction

Difficult biliary cannulation is encountered in approximately 20–30% of endoscopic retrograde cholangiopancreatography (ERCP) procedures, increasing the risk of adverse events [1,2]. Selecting an appropriate cannulation method can reduce the incidence of adverse events in these patients. When simple cannulation fails, advanced techniques are employed to facilitate deep biliary access. Following repeated inadvertent pancreatic duct cannulation, the European Society of Gastrointestinal Endoscopy (ESGE) guidelines recommend considering pancreatic guidewire-assisted (PGW) techniques as the preferred approach [1]. However, selection of the cannulation method greatly varies among endoscopists, and needle-knife precut methods are frequently preferred over PGW-assisted methods. The double-guidewire technique (DGW) [3], transpancreatic sphincterotomy (TPS) [4], and pancreatic stent-assisted cannulation (PPS-C) [5] are the most commonly used options among the PGW-assisted methods. TPS has been demonstrated to be a very effective and safe advanced cannulation method in previous meta-analyses [6,7,8], and TPS was even considered superior to other advanced methods. However, only a few high-quality randomized controlled trials (RCTs) have compared TPS with other advanced cannulation methods [9,10]. In this study, we provide real-world data on the complexities of PGW-assisted advanced cannulation strategies and further salvage methods in case of cannulation failure used in Hungarian tertiary centers and compare the outcomes of different techniques. By comparing the effectiveness and safety of these techniques, we seek to inform clinical practice and optimize the management of difficult biliary cannulation in ERCP.

## 2. Materials and Methods

### 2.1. General Considerations

The Hungarian ERCP Registry (H-ERCP), initiated in 2016 by the Hungarian Endoscopy Study Group and the Institute for Translational Medicine, University of Pécs, provided data from 7 tertiary referral centers and 18 endoscopists for this analysis [11]. All participating endoscopists had high yearly and lifetime case numbers. Consecutive patient enrollment was expected. All cases between September 2016 and December 2020 were included, with 30-day follow-up conducted to detect late adverse events. A rigorous four-step quality check system ensured accurate data collection (1: local check from administrator, 2: endoscopist, 3: central check by chief administrator, 4: registry coordinator). This study was approved by the Scientific and Research Ethics Committee of the Medical Research Council (by the approval codes of TUKEB-35523/2016/EKU and BMEÜ/714-1/2022/EKU). Informed consent was obtained from all participants in this prospective study. This cohort study conforms to the STROBE guidelines [12] (checklist available in the Supplementary Materials, Supplementary Table S2).

### 2.2. Inclusion and Exclusion Criteria

**Figure 1:**
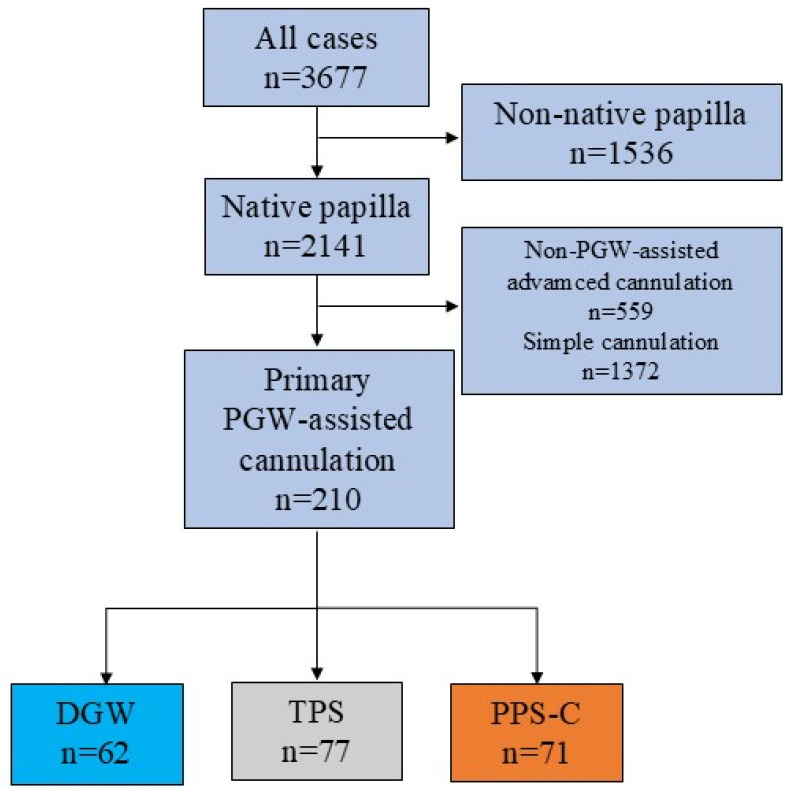
Flowchart of the case selection of primary pancreatic guidewire-assisted cannulation methods (PGW: pancreatic guidewire, DGW: double guidewire technique, TPS: transpancreatic sphincterotomy, PPS-C: prophylactic pancreatic stent-assisted cannulation, light blue box: DGW, grey box: TPS, orange box: PPS-C).

From the 3677 available ERCP cases, we selected native papilla cases with biliary indications. Of these, 234 cases involved PGW-assisted cannulation (DGW, TPS, PPS-C), either as the primary technique (n = 210) or as a rescue method (n = 24) after failed non-PGW-assisted attempts (Figure 1). Cases were excluded if a non-PGW-assisted method was used or if non-native papilla was present. Post-ERCP pancreatitis (PEP) could not be evaluated when the indication of ERCP was acute biliary pancreatitis (ABP), so these cases were excluded from the analysis of this adverse event.

### 2.3. Definitions

Indications and complications were classified according to American Society of Gastrointestinal Endoscopy (ASGE) and ESGE guidelines [13,14].

Primary cannulation attempts with a PGW-assisted advanced cannulation method (DGW, TPS, and PPS-C) means that these techniques were applied first after unsuccessful conventional guidewire cannulation attempts. Secondary and tertiary cannulation attempts (salvage advanced cannulation) were classified as follows: after failed attempts of cannulation with the primary PGW-assisted advanced cannulation method, a secondary—and after failure of that, in some cases, a third—advanced cannulation method was used.

### 2.4. Analyzed Dataset

Demographic data (sex, age, American Society of Anesthesiologists [ASA] score), indications for ERCP, deep biliary cannulation success rate, cannulation and fluoroscopy times, and complication rates were analyzed. The use of post-ERCP pancreatitis prophylaxis (non-steroidal anti-inflammatory drug suppositories and prophylactic pancreatic stents) was also evaluated. The objective difficulty of ERCP was also compared using the ASGE score [15]. The data quality was good, and missing values were rare or absent for most outcomes. Patients with missing data were excluded from the analysis. As the outcomes did not require a long follow-up period, no attrition was appreciated.

### 2.5. Statistical Analysis

Continuous variables were calculated and are presented as means and standard deviations (SDs) or medians and interquartile ranges (IQRs). Categorical data are presented as percentages. The Kruskal–Wallis rank sum test was used to observe differences between the groups. The chi-square test or Fisher’s exact test was used to analyze the relationships between categorical variables. All the analyses were performed using the R statistical software (R Core Team (2023). R: Language and environment for statistical computing. R Foundation for Statistical Computing, Vienna, Austria).

## 3. Results

### 3.1. General Characteristics of the Cohort

Altogether, 3677 ERCP cases in the registry at the time of analysis were included. Of the 2142 native papilla cases, 234 cases of PGW-assisted advanced cannulation were analyzed. The primary PGW-assisted technique selected was TPS in 77 cases, DGW in 62 cases, and PPS-C in 71 cases. No significant differences in the average age, body mass index, presence of juxtapapillary diverticula, or ASA scores were observed between the groups; however, the PPS-C group comprised significantly fewer female patients than the other groups (Supplementary Table S1).

### 3.2. Indications of ERCP

The most common indications for ERCP are biliary tract disease, obstructive jaundice, acute cholangitis, and biliary pancreatitis. PPS-C was used more frequently in patients with acute biliary pancreatitis than the other techniques (*p* = 0.024) (Supplementary Figure S1).

### 3.3. Objective Grading of ERCP Difficulty

TPS was used most often in grade 3 difficulty cases (47% of all TPS cases), whereas only 27.4% of DGW cases were grade 3 (*p* = 0.02). Most cases were grades 2 and 3, indicating a higher proportion of complex cases in this tertiary center cohort (Supplementary Figure S2).

### 3.4. Biliary Cannulation Success Rates

**Figure 2:**
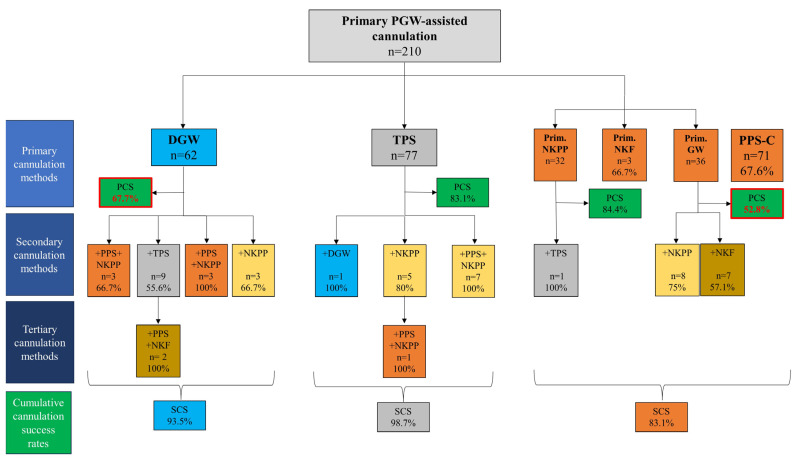
Flowchart of the primary pancreatic guidewire-assisted cannulation methods (PCS: primary cannulation success rate, SCS: secondary cannulation success rate, DGW: double guidewire technique, TPS: transpancreatic sphincterotomy, PPS-C: prophylactic pancreatic stent-assisted cannulation, NKPP: conventional needle-knife precut papillotomy, NKF: needle-knife fistulotomy, GW: guidewire-assisted cannulation, PGW: pancreatic guidewire, light blue box: DGW, grey box: TPS, orange box: PPS-C, yellow box: NKPP, brown box: NKF). The cannulation rates of the least successful methods are highlighted in red.

The primary cannulation success rate was highest with TPS (83.1%), significantly higher than DGW (67.7%) and PPS-C (67.6%) (*p* < 0.001). In the PPS-C group, subsequent needle-knife precut papillotomy (NKPP) achieved a high cannulation success rate (84.4%). In contrast, PPS-assisted guidewire cannulation resulted in a significantly lower success rate (52.8%). With salvage advanced methods, cannulation success reached 98.7% in the TPS group, while it was only 93.5% in DGW, and 83.1% in PPS-C group (*p* = 0.006) (Table 1, Figure 2).

### 3.5. Salvage Advanced Cannulation Methods Used After Failed PGW-Assisted Cannulation

In most cases where DGW failed as the primary approach (n = 18), TPS was the most frequently chosen salvage technique (n = 9). Despite its theoretical advantages, TPS in this setting demonstrated a modest success rate of 55.6%. Interestingly, a small subset of patients (n = 3) who underwent PPS implantation followed by needle-knife papillotomy (NKPP) as a salvage strategy after DGW achieved a 100% success rate, suggesting a potentially promising approach in this specific scenario.

When TPS was unsuccessful as the primary method, salvage attempts proved remarkably effective, with a 98.7% overall success rate. Notably, DGW was rarely employed as a secondary technique (n = 1, successful), indicating a preference for alternative approaches like NKPP (n = 5, 80% success) or a combination of PPS insertion followed by NKPP (n = 7, 100% success). This high success rate after failed TPS highlights the potential for successful biliary cannulation with appropriate salvage techniques, even after the initial TPS has been unsuccessful.

In cases where PPS-C was unsuccessful as the primary method, subsequent guidewire-assisted cannulation proved to be the least effective salvage technique, with a success rate of only 52.8%. Additionally, salvage attempts with NKPP (75% success) or needle-knife fistulotomy (NKF) (57.1% success) yielded relatively low success rates. In contrast, NKPP after PPS demonstrated a high cannulation success rate (87.4%), suggesting that this approach may be more suitable in cases of PPS-C. Interestingly, secondary TPS after primary NKPP PPS-C cannulation also showed efficacy (Figure 2).

### 3.6. Adverse Event Rates

The overall rate of adverse events was low, with no statistically significant differences between groups. The highest post-ERCP pancreatitis (PEP) rate was observed in the DGW group (5.6%), but this did not reach statistical significance (*p* = 0.923) (Table 2).

### 3.7. Post-ERCP Pancreatitis Prophylaxis

Indomethacin suppository was used in most patients in the DGW group (93.5%), whereas it was not frequently used in the TPS (53.2%) and PPS-C groups (33.8%). PPS use was also low in the TPS (61.0%) and DGW groups (56.5%) despite the recommendations (Table 3).

Of the 10 cases of PEP, 3 occurred after DGW, all of whom received indomethacin but no PPS. Two cases of PEP received no prophylaxis with either a non-steroid suppository or a prophylactic stent in the TPS and TPS + DGW groups (Table 4).

### 3.8. Cannulation and Fluoroscopy Times

The time required to achieve deep biliary cannulation did not differ significantly between the groups; however, the median cannulation time in the TPS group was 7 min (420 s), which was 1.4 times longer than that in the PPS-C group (300 s).

The longest fluoroscopy time was measured in the DGW group (185 s), which was more than a minute longer than that in the other two groups (*p* = 0.047) (Table 5).

### 3.9. PGW-Assisted Cannulation Methods as Salvage Advanced Cannulation Techniques

PGW-assisted cannulation was rarely used as a salvage technique after failed non-PGW-assisted attempts (n = 24).

**Figure 3:**
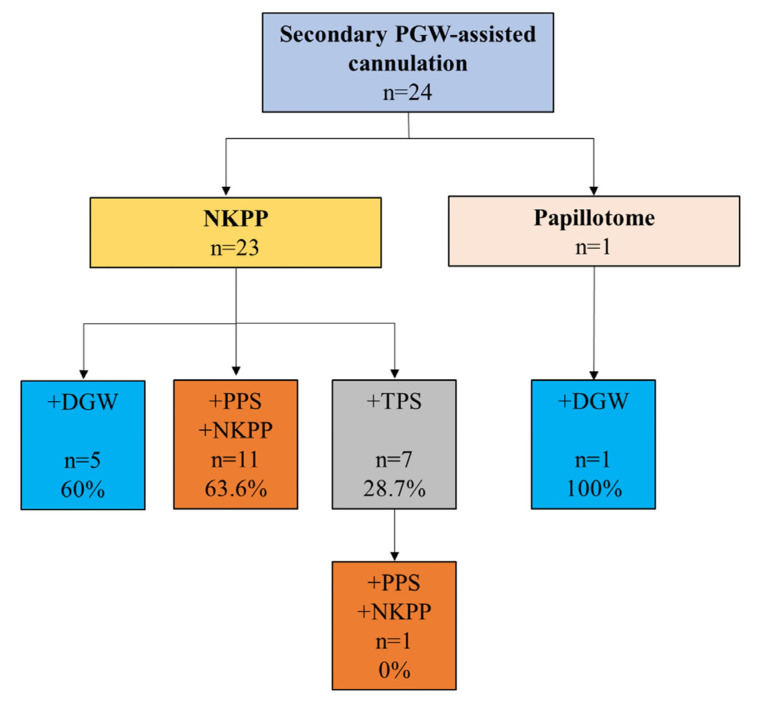
Flowchart of the secondary pancreatic guidewire-assisted cannulation methods and their success rates after non-PGW-assisted methods (DGW: double guidewire technique, TPS: transpancreatic sphincterotomy, PPS: prophylactic pancreatic stent, NKPP: conventional needle-knife precut papillotomy, light blue box: DGW, grey box: TPS, orange box: PPS-C, yellow box: NKPP, pink box: papillotome).

The cannulation success rate was really low (28.7% in NKPP + TPS, 60.0% in NKPP + DGW, 63.6% in NKPP + PPS + NKPP group) (Figure 3). Two cases of PEP and one case of late bleeding occurred after salvage cannulation. No perforations were observed in any patient.

## 4. Discussion

In this study, we report real-world data from a prospectively collected registry on the PGW-assisted advanced cannulation method using outcomes from seven high-volume centers in Hungary.

The overall cannulation success rate was high, and the rate of adverse events was low. The biliary cannulation was significantly higher in the TPS group, which is in line with previous reports. However, the recommended prophylactic methods for PEP have not been applied in a considerable proportion of cases. Some recent studies have not used routine PPS after TPS [10] despite current recommendations [1,13]. PPS is a straightforward and easily applicable approach, because the guidewire is already deeply inserted into the pancreatic duct during TPS or DGW cannulation. Theoretically, the resistance of pancreatic outflow decreases after TPS because the pancreatic sphincter is partially or fully cut; therefore, much less benefit can be expected from PPS in this situation. However, rigorous comparative studies need to be conducted to verify this [16]. The average PEP rate after TPS was 7.1% in our previous meta-analysis, in which most of the included studies did not apply PPS [6]. Different studies have demonstrated great variability in PEP rates after TPS. Barakat et al. have reported a PEP rate as low as 1.1%, whereas PPS was used in only 11.1% of the TPS cases [17]. No significant difference in the rate of post-ERCP pancreatitis was observed between patients in each group who underwent PPS and those who did not. Kylänpää et al. have reported unusually high PEP rates, namely 13.5% after TPS and 16.2% after DGW, where the PPS usage was similarly low in both groups (8.7% and 11.1%, respectively) [10]. By contrast, another randomized study compared DGW and TPS, in which all patients received PPS, and the PEP rate was only 2.9% in both groups [9].

TPS and PPS-C with primary needle-knife papillotomy had the highest success rates, whereas DGW had a low primary cannulation success rate. PPS-C with subsequent GW cannulation attempts leads to frequent cannulation failures, even with further salvage methods. Therefore, further precutting is advised after PPS insertion to achieve selective biliary cannulation. TPS usage after NKPP did not achieve an acceptable cannulation rate; therefore, we do not recommend it in these cases. DGW and PPS + NKPP provided better cannulation outcomes. After failed DGW, TPS does not seem to be the best approach with lower rates of successful biliary cannulation as needle-knife techniques, as supported by new RCT results, too [18]. After failed TPS, NKPP and PPS + NKPP resulted in high rates of successful biliary access and did not result in increased adverse outcomes in this cohort.

Free-hand needle-knife techniques could also be used in these situations; however, when the pancreatic duct is cannulated, a PGW-assisted method should be tried as the first-line method according to the ESGE cannulation algorithm as it provides more stability and easy access to prophylactic pancreatic stent placement [13].

The numerically highest rate of PEP was observed in the DGW group, which is consistent with previous findings [19]. Contrary to the recommendations, none of the patients who developed PEP received PPS. New data from post hoc analyses of an RCT supports the combined use of PPS and indomethacin and could be protective even after a high number of inadvertent pancreatic guidewire passages [20]

The use of the appropriate cannulation method is of crucial importance in cases of acute biliary pancreatitis patients, where according to our previously published study, higher rates of advanced cannulation technique use and also higher rates of pancreatic duct cannulation were seen. In these patients, we hypothesize that pancreatic duct stenting and also the appropriate use of PGW-assisted methods could lead to better outcomes [21,22,23].

TPS has been demonstrated to be a very effective and safe advanced cannulation method in previous meta-analyses [6,7,8], and TPS was even considered superior to other advanced methods. However, only a few randomized controlled trials (RCTs) have compared TPS with other advanced cannulation methods. A meta-analysis performed in 2021 selected only RCTs comparing TPS and DGW and identified that TPS resulted in successful biliary access in 93.3% of cases, whereas DGW was successful in only 79.4% of cases; however, the difference was not statistically significant. By contrast, a significantly lower PEP rate was observed with TPS (8.9% vs. 22.2%) [24] In recent randomized studies, TPS resulted in a significantly higher biliary cannulation rate than DGW [9,10].

TPS is an infrequently used advanced cannulation method compared to more widespread and potentially more dangerous needle-knife precut techniques, which require a high level of endoscopic skill. Needle-knife precut is a freehand technique, whereas TPS and other PGW-assisted techniques are led by a guidewire and allow better control. TPS has also been indicated as easier to learn than other advanced cannulation methods. In a large retrospective series, the TPS technique was considered straightforward for self-learning [17]. Previous studies of TPS were performed in high-volume centers by experienced endoscopists, although newer evidence revealed that non-expert endoscopists also benefit from utilizing TPS, which can significantly improve their cannulation rate and enhance the quality of ERCP, irrespective of practice volume [25]. The long-term safety of TPS has also been evaluated with a follow-up period of four to ten years. No additional risk of chronic pancreatitis and abdominal pain was demonstrated [26]. In regard to anatomical factors, the papilla morphology could also potentially impact the success of TPS and other techniques, as demonstrated in several studies, which should also be taken into account when choosing the appropriate cannulation method [27,28].

### 4.1. Strengths and Limitations

This study has several strengths. Here, we have presented a large number of cases and prospectively collected registry data from seven Hungarian tertiary centers. Our ERCP registry has multiple built-in quality checks that limit incorrect data entry and underreporting. We present complete and detailed data on the complexities of different advanced cannulation strategies and salvages methods.

Nevertheless, this study also has some limitations. All participating hospitals and endoscopists received a high volume of patients, but the case distribution varied among centers and endoscopists, thus hindering generalizability. All endoscopists who participated are working in high-volume centers and managing high yearly case numbers. The number of cannulation attempts was not recorded. The radiation dose could not be calculated; instead, only the fluoroscopy time was recorded in the registry. The adverse event rate was low, and for that reason, this study was underpowered to detect any meaningful differences, and it required much larger sample sizes, but showed the general safety of these methods. Additionally, reliable multivariate analyses could not be executed because of the low event numbers, and for this reason, the effects of confounders on the results could not be excluded.

### 4.2. Implications for Practice and Research

In practice, transpancreatic sphincterotomy (TPS) is a viable alternative to other pancreatic guidewire-assisted methods, with a high cannulation success rate and low rate of adverse events. Based on our data, TPS works well in non-disrupted anatomy; however, it is not recommended as a sequential cannulation method after failed needle-knife precut papillotomy (PPS-NK).

Randomized controlled trials should be conducted to examine the effectiveness of the PPS-GW and PPS-NK approaches.

## 5. Conclusions

In conclusion, TPS and PPS-C with a primary needle-knife precut have high cannulation success rates and low adverse event rates, whereas DGW requires a higher rate of salvage techniques. Based on our data, DGW cannulation is not recommended for biliary cannulation because of its low success rate. TPS is also not recommended as a secondary advanced cannulation method after failed NKPP because the biliary access rate was very low in this setting. In all cases of PGW-assisted cannulation, a PPS should be placed to minimize the risk of PEP. The efficacy and safety of different PGW-assisted advanced cannulation methods should be further examined in randomized controlled trials and registry-based prospective comparative studies with standardized prophylaxis protocols.

## Figures and Tables

**Table 1:** Biliary cannulation success rates according to primary PGW-assisted cannulation groups (DGW: double guidewire technique, TPS: transpancreatic sphincterotomy, PPS-C: prophylactic pancreatic stent-assisted cannulation).

Primary Cannulation Method	TPS (n = 77)	DGW (n = 62)	PPS-C (n = 71)	*p*-Value
Primary cannulation success rate	64 (83.1%)	42 (67.7%)	48 (67.6%)	<0.001
Secondary cannulation success rate	76 (98.7%)	58 (93.5%)	59 (83.1%)	0.006

**Table 2:** Adverse event rates in the primary PGW-assisted cannulation groups (DGW: double guidewire technique, TPS: transpancreatic sphincterotomy, PPS-C: prophylactic pancreatic stent-assisted cannulation, N/A: not applicable). Acute biliary pancreatitis (ABP) cases were excluded from analysis in case of post-ERCP pancreatitis rate.

	TPS (n = 77)	DGW (n = 62)	PPS-C (n = 71)	*p*-Value
Post-ERCP pancreatitis (ABP cases excluded)	3/59 (5.0%)	3/54 (5.6%)	2/51 (3.9%)	0.923
Clinically significant bleeding	1 (1.3%)	0 (0.0%)	2 (2.8%)	0.838
Immediate bleeding	8 (11%)	12 (19.4%)	15 (21.1%)	0.184
Perforation	0 (0.0%)	0 (0.0%)	0 (0.0%)	N/A
Cholangitis	1 (1.3%)	0 (0.0%)	3 (4.2%)	0.267
Cholecystitis	0 (0.0%)	1 (1.6%)	0 (0.0%)	0.293

**Table 3:** Post-ERCP pancreatitis prophylaxis use in the PGW-assisted cannulation method cohort (DGW: double guidewire technique, TPS: transpancreatic sphincterotomy, PPS-C: prophylactic pancreatic stent-assisted cannulation).

	TPS (n = 77)	DGW (n = 62)	PPS-C (n = 71)	*p*-Value
Indomethacin suppository use	41 (53.2%)	58 (93.5%)	24 (33.8%)	<0.001
PPS use	47 (61.0%)	35 (56.5%)	71 (100.0%)	<0.001
No PEP prophylaxis used	25 (32.5%)	0 (0%)	0 (0%)	<0.001

**Table 4:** Post-ERCP pancreatitis cases and prophylaxis methods use in the cohort (DGW: double guidewire technique, GW: guidewire, TPS: transpancreatic sphincterotomy, PPS-C: prophylactic pancreatic stent-assisted cannulation, NKPP: conventional needle-knife precut papillotomy, PPS: prophylactic pancreatic stent).

Post-ERCP Pancreatitis Cases by Groups		n	PPS	Indomethacin Suppository
Primary PGW-assisted groups	DGW	3	0/3 (0%)	3/3 (100%)
	PPS + GW	1	1/1 (100%)	1/1 (100%)
	PPS + NKPP	1	1/1 (100%)	0/1 (0%)
	TPS	2	1/2 (50%)	0/2 (0%)
	TPS + DGW	1	0/1 (0%)	0/1 (0%)
Salvage PGW-assisted groups	NKPP + TPS	1	0/1 (0%)	1/1 (100%)
	NKPP + PPS + NKPP	1	1/1 (100%)	1/1 (100%)

**Table 5:** Cannulation and fluoroscopy times in cases of TPS, DGW, and PPS-C cannulation (DGW: double guidewire technique, TPS: transpancreatic sphincterotomy, PPS-C: prophylactic pancreatic stent-assisted cannulation).

	TPS (n = 77)	DGW (n = 62)	PPS-C (n = 71)	*p*-Value
Cannulation time (median—IQR) in seconds	420 (120, 627)	335 (240, 602)	300 (120, 606)	0.179
Fluoroscopy time (median—IQR) in seconds	100 (80, 143)	185 (108, 256)	110 (65, 172)	0.047

## Data Availability

All relevant data are presented in the paper and Supporting Information files. Additional data are available upon request from the corresponding author.

## Supplementary Materials

The following supporting information can be downloaded at https://www.mdpi.com/article/10.3390/jcm14197118/s1, Figure S1: Distribution of indications of ERCP; Figure S2: Objective grading of ERCP difficulty; Table S1: General characteristics of the cohort;.Table S2: STROBE Statement—checklist of items that should be included in reports of observational studies.

## Abbreviations

The following abbreviations are used in this manuscript:

ABP	acute biliary pancreatitis
ASA	American Society of Anesthesiologists
ASGE	American Society of Gastrointestinal Endoscopy
DGW	double-guidewire technique
ERCP	endoscopic retrograde cholangiopancreatography
ESGE	European Society of Gastrointestinal Endoscopy
IQR	interquartile range
NKPP	needle-knife papillotomy
PEP	post-ERCP pancreatitis
PGW	pancreatic guidewire
PPS-C	prophylactic pancreatic stent-assisted cannulation
SD	standard deviation
TPS	transpancreatic sphincterotomy
